# New Concept of Polymethyl Methacrylate (PMMA) and Polyethylene Terephthalate (PET) Surface Coating by Chitosan

**DOI:** 10.3390/polym8040132

**Published:** 2016-04-07

**Authors:** Mieszko Wieckiewicz, Eric Wolf, Gert Richter, Heike Meissner, Klaus Boening

**Affiliations:** 1 Division of Dental Materials, Faculty of Dentistry, Wroclaw Medical University, 26 Krakowska st., 50425 Wroclaw, Poland; 2 Department of Prosthetic Dentistry, Faculty of Medicine, Dresden University of Technology, Fetscherstrasse 74, 01307 Dresden, Germany; erpawolf@aol.com (E.W.); e-g-richter@gmx.de (G.R.); Heike.Meissner@uniklinikum-dresden.de (H.M.); klaus.boening@uniklinikum-dresden.de (K.B)

**Keywords:** PMMA, PET, chitosan, adhesion, coating

## Abstract

Chitosan is known for its hemostatic and antimicrobial properties and might be useful for temporary coating of removable dentures or intraoral splints to control bleeding after oral surgery or as a supportive treatment in denture stomatitis. This study investigated a new method to adhere chitosan to polymethyl methacrylate (PMMA) and polyethylene terephthalate (PET). There were 70 cylindrical specimens made from PMMA and 70 from PET (13 mm diameter, 6 mm thickness). The materials with ten specimens each were sandblasted at 2.8 or 4.0 bar with aluminum oxide 110 μm or/and aluminum oxide coated with silica. After sandblasting, all specimens were coated with a 2% or 4% acetic chitosan solution with a thickness of 1 mm. Then the specimens were dried for 120 min at 45 °C. The precipitated chitosan was neutralized with 1 mol NaOH. After neutralization, all specimens underwent abrasion tests using the tooth-brushing simulator with soft brushes (load 2N, 2 cycles/s, 32 °C, 3000 and 30,000 cycles). After each run, the specimen surfaces were analyzed for areas of remaining chitosan by digital planimetry under a light microscope. The best chitosan adhesion was found after sandblasting with aluminum oxide coated with silica (*U*-Test, *p* < 0.05) in both the PMMA and the PET groups. Hence, with relatively simple technology, a reliable bond of chitosan to PMMA and PET could be achieved.

## 1. Introduction

Chitosan (CS) is an amino-polysaccharide chain obtained in the reaction of deacetylation of chitin [1]. Its primary source is crustaceans. Its structure is based on repetitive d-glucosamine units linked with fewer, randomly distributed *N*-acetyl-d-glucosamine units by a β-1,4-glycosidic bond [1,2]. CS is formed during the process of *N*-deacetylation of chitin, which involves the disruption of acetamide bonds in order to remove the acetyl group [3]. Chitosan is a non-toxic, biodegradable, biofunctional, and biocompatible compound with hydrophilic properties [4,5]. It shows both chemoattractant and antimicrobial activity and has the ability to activate macrophages and neutrophils. It stimulates cellular activity of, for example, fibroblasts, as well as detects growth factors, stimulates the production of cytokines and collagen, and promotes the processes of angiogenesis [6]. These characteristics allow CS to promote the process of coagulation and wound healing and stimulate the formation of granulation tissue and re-epithelialization. It should be emphasized that CS has many reactive functional groups, which are responsible for the high chemical activity of this substance [7]. For specific medical and pharmaceutical applications, CS is available in different degrees of deacetylation as well as different molecular weights. Furthermore, CS can easily be modified chemically and enzymatically.

Numerous applications of CS in medicine have been described. However, little attention is being paid to the potential benefits of CS in wound treating and tissue management in the oral cavity, for instance after oral surgery or in cases of denture stomatitis. According to the literature, the latter one has a prevalence of 15% to 70% in removable dental prostheses wearers [8,9]. To serve the purpose as an adjuvant therapeutic agent for the indications mentioned above it might be appropriate to use surgical splints or denture bases as carriers to apply CS to the oral mucosa. The difficulty of this method is to achieve an abrasion-resistant bond between the hydrophilic CS and intraoral devices made from more hydrophobic resins such as polymethyl methacrylate (PMMA) and polyethylene terephthalate (PET). PMMA has long been used in restorative dentistry and can be considered a first-choice resin base material in removable dentures. Also, PET has a wide application in dentistry in, for example, orthodontic retainers, mouthguards, bite splints in patients with bruxism or protective splints after oral surgery. It is cost effective and can easily be molded by thermoforming in the dental laboratory.

The aim of the study was to develop a reliable, reproducible and cost-effective method to bond CS to PMMA or PET using special dental sandblasting equipment (Rocatector Delta sandblaster, 3M ESPE, Seefeld, Germany) in order to achieve an abrasion-resistant chitosan coating for surgical splints and removable dentures. Standard application of the Rocatector sandblaster in dental technology is a tribo-chemical silicate coating of dental porcelain, metals and resins by sandblasting with silica-modified aluminum oxide. Each particle of the sand is coated with a thin layer of SiO_2_. Components of silica-modified particles (Rocatec Plus blasting agent, 3M ESPE, Seefeld, Germany) are retentively anchored on the surface due to their high impact energy creating a hydrophilic adhesive layer (silicate coating). Contrary to the experiments in this study, where chitosan was applied directly after sandblasting, the original procedure subsequently applies an organic silane coupling agent to achieve a chemical bond to dental adhesives [10,11].

Abrasion tests were performed to evaluate the quality of the bond of CS to PMMA and PET for short-term use (several days) as well as a potential long-term use (several weeks).

## 2. Experimental Section

### 2.1. Materials

**Figure 1:**
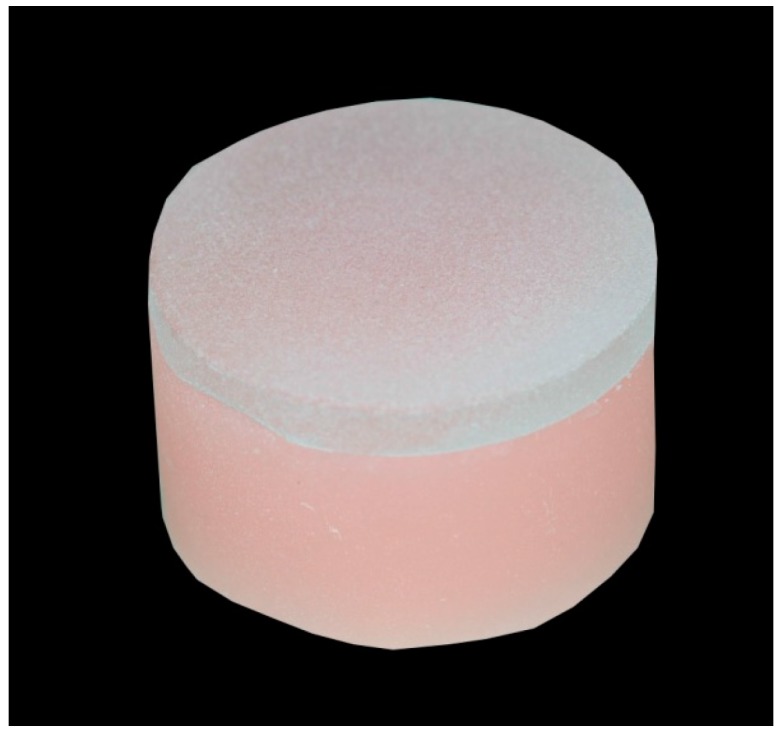
Specimen with PET surface.

For the experiments, a total of 140 cylindrical specimens were made from PMMA (Palapress, Heraeus-Kulzer, Hanau, Germany). The specimens were prepared according to the manufacturer’s instructions, *i.e.*, the powder-to-liquid ratio was 10 g to 7 mL, mixed for 15 s at room temperature (approx. 23 °C), poured into the casting mold within a period of two minutes, polymerized for 20 min at 55 °C and a pressure of 2.5 bar and smoothed with 1000 grit sandpaper. There were 70 PMMA specimens of 13.0 mm diameter and 6.0 mm height; a further 70 specimens were 13.0 mm in diameter and 5.0 mm in height. For the latter ones, 70 PET disks of 13.0 mm in diameter and 1.0 mm in height (Erkodur, ERKODENT Erich Kopp GmbH, Pfalzgrafenweiler, Germany) were prepared and glued to the PMMA cylinder’s front surfaces using quick-setting cyanoacrylate glue (Sekundenkleber, Renfert GmbH, Hilzingen, Germany). The specimens were divided into 14 groups with 10 specimens each: 7 groups to investigate the abrasion resistance between PMMA and CS, and 7 groups to investigate the abrasion resistance between PET and CS (Figure 1).

This study used chitosan 90/500 (degree of deacetylation 87.6%–92.5%, 200–400 kDa, Heppe Medical Chitosan GmbH, Halle, Germany). Two different CS solutions were prepared both using 2% acetic acid obtained from the hospital pharmacy of the Carl Gustav Carus University Hospital (Dresden University of Technology, Dresden, Germany): one solution containing 2% CS, and one solution containing 4% CS. The CS was dissolved at a temperature of 60 °C using a magnetic stirrer (RET CV S000, IKA, Staufen, Germany). After preparation, the solutions were stored in a refrigerator at 6 °C.

The Rocatector Delta sandblaster (3M ESPE, Seefeld, Germany) with the microblasting agents Rocatec Pre (aluminum oxide, grain size 110 μm) and Rocatec Plus (aluminum oxide, grain size 110 μm with each grain being silica-coated) was used for cleaning (Rocatec Pre, Seefeld, Germany) and silicate coating (Rocatec Plus, Seefeld, Germany) of the PMMA and PET surfaces prior to applying acetic CS solution.

### 2.2. Methods

**Figure 2:**
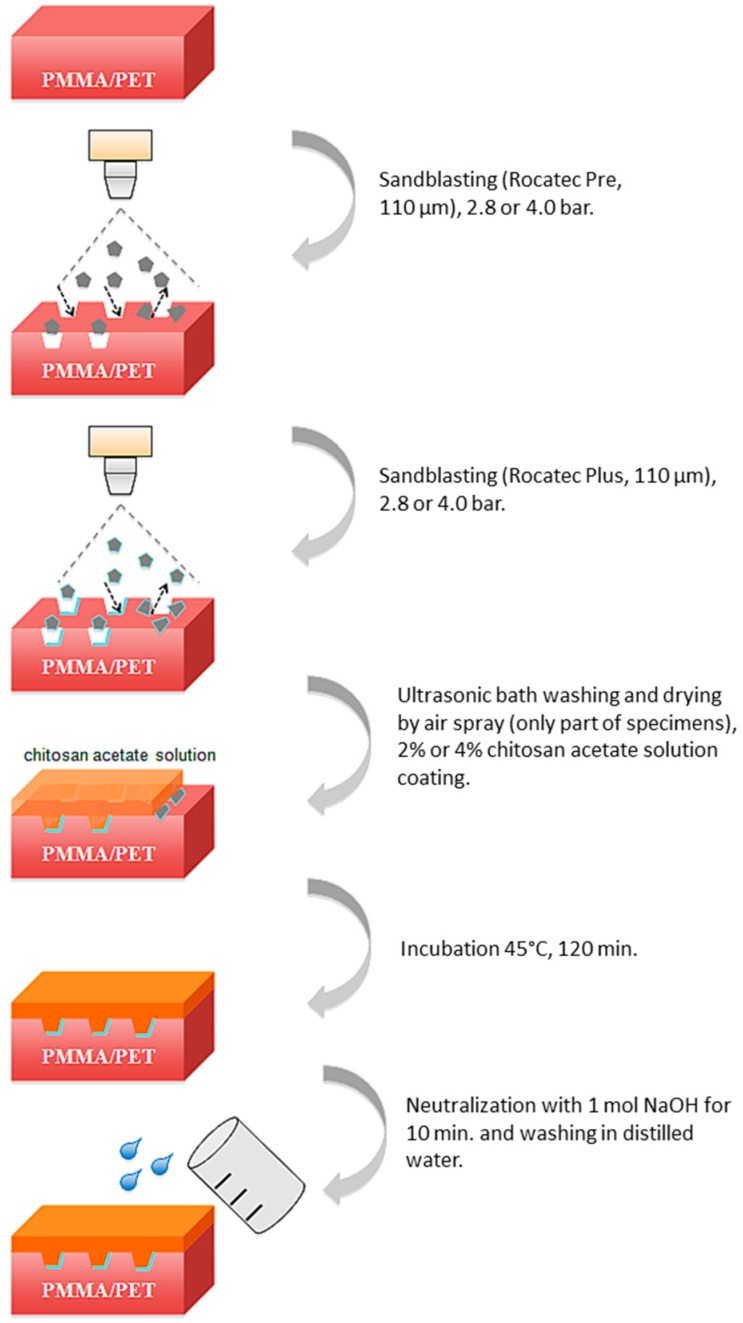
Illustration of workflow to coat resin specimens with CS.

**Figure 3:**
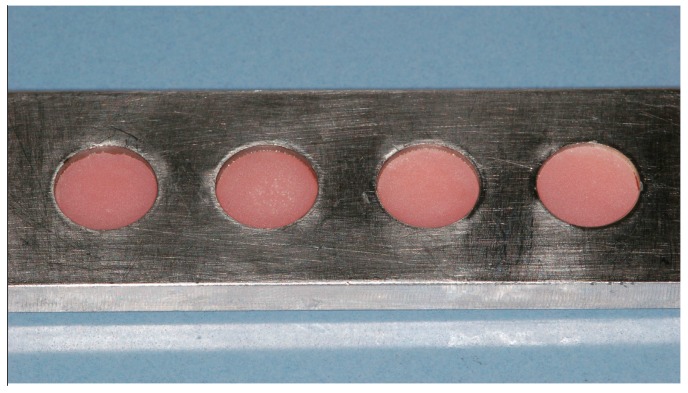
Template for coating the specimens with acetic CS solution.

The basic procedure to coat the specimens with CS was as follows (Figure 2):

1. Sandblasting of the surfaces to be CS coated at a distance of 20–30 mm with Rocatec Pre and Rocatec Plus.
2. Washing in an ultrasonic bath (distilled water) for 10 min to remove loose grains from sandblasting and drying with an air spray.
3. Coating with acetic CS solution of 1 mm thickness using a metal template (Figure 3).
4. Drying the CS solution in an incubator (B6030, Heraeus, Hanau, Germany) for 120 min at 45 °C.
5. Neutralization of the precipitated CS in 1 mol NaOH solution for 10 min.
6. Washing in distilled water and storing dry at room temperature until further use.

The following variations within steps 1, 2 and 3 of the basic procedure were examined with the intention to optimize the bond between CS and PMMA or PET. Within step 1 of the basic procedure, three variations (a, b and c) were examined:

- sandblasting with Rocatec Pre only (10 s, 2.8 bar) or
- sandblasting with Rocatec Pre (10 s, 2.8 bar) and sandblasting with Rocatec Plus (13 s, 2.8 bar) or
- sandblasting with Rocatec Pre (10 s, 2.8 bar) and sandblasting with Rocatec Plus (13 s, 4.0 bar)

Within step 2 two variations, a and b, were examined:

- washing in an ultrasonic bath
- omitting the step of washing in an ultrasonic bath

Within step 3, the following two variations, a and b, were examined:

- coating with acetic CS solution containing 2% chitosan or
- coating with acetic CS solution containing 4% chitosan

Combing these variations of steps 1 to 3 of the basic procedure resulted in a sequence of seven test series listed in Table 1. All procedures were performed on PMMA and on PET surfaces.

**Figure 4:**
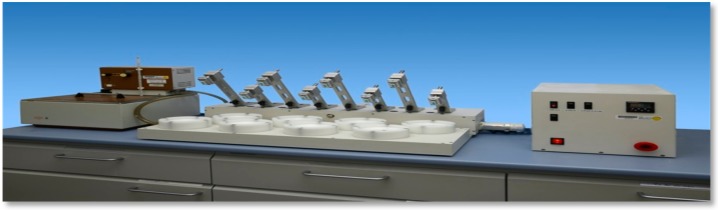
Willytec tooth-brushing simulator for abrasion tests.

After CS coating all specimens underwent abrasion tests using a Willytec tooth-brushing simulator (Willytec GmbH, Munich, Germany) with soft brushes in an artificial saliva (Table 2) (load 2N, 2 cycles/s, 32 °C, Figure 4). To simulate short-term use, 3000 cycles of abrasion were performed on all CS-coated specimen surfaces. To simulate long-term use, all remaining CS coatings were removed, the specimen surfaces smoothed again with 1000 grit sandpaper, the CS coatings renewed, and all specimen surfaces exposed to 30,000 cycles of abrasion.

**Figure 5:**
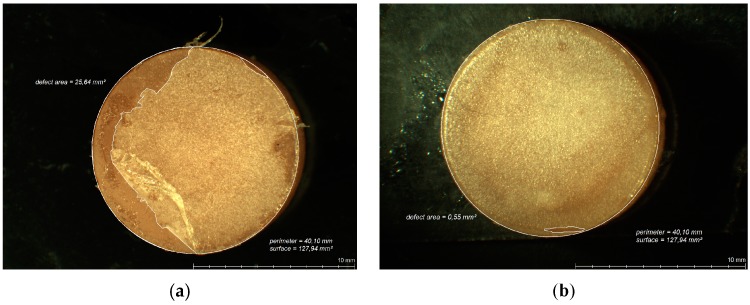
Light microscopic analysis of remaining CS on PET specimens after 30,000 cycles of abrasion by simulated tooth brushing ((**a**) taken from test series I; (**b**) taken from test series VI).

**Figure 6:**
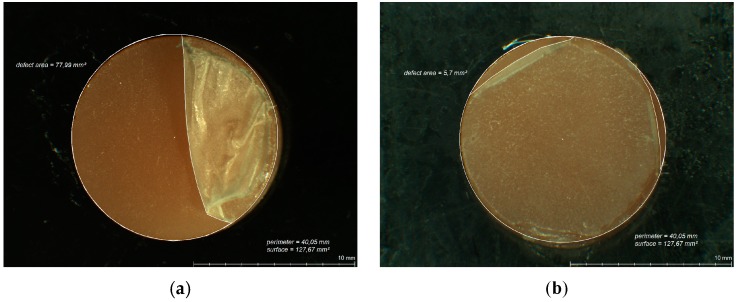
Light microscopic analysis of remaining CS coating on PMMA specimens after 30,000 cycles of abrasion by simulated tooth brushing ((**a**) taken from test series I; (**b**) taken from test series VI).

After each abrasion test, the specimen surfaces were analyzed for areas of lost CS coating (Figure 5a,b and Figure 6a,b) by means of digital planimetry under a light microscope (Leica MZ12, Meyer Instruments, Houston, TX, USA) and the percentage of remaining intact CS coating calculated.

### 2.3. Hypotheses and Statistical Analysis

The modifications of steps 1, 2 and 3 in the basic procedure (*i.e.*, test series I to VII in Table 1) were carried out with the aim to optimize an abrasion-resistant bond of CS to PET or PMMA. The following null hypotheses were stated:

1. Modifying the sandblasting procedure (using/omitting Rocatec Plus) does not influence the bond of CS to PMMA or PET
2. The CS concentration in the acetic solution (2% or 4%) does not influence the bond of CS to PMMA or PET
3. The blast pressure (2.8 or 4.0 bar) does not influence the bond of CS to PMMA or PET
4. Omitting the step of ultrasonic cleaning does not influence the bond of CS to PMMA or PET
5. PMMA and PET are equally suitable for bonding CS to their surfaces.

Given a number of 10 specimens in each group, cases of the lack of normal distribution and a lack of homogeneity of variance the non-parametric, the Mann-Whitney *U*-test was thus chosen for data analysis (STATISTICA software, version 10, StatSoft Inc., Tulsa, OK, USA). The statistical significance level was set as *p* = 0.05. However, since the experiment involved multiple comparisons, the alpha level was Bonferroni corrected. Depending on the experimental condition, the number of multiple comparisons was between 2 and 4. In the study, we used a conservative approach and corrected significance alpha level for four comparisons globally. Consequently, only if the *U*-test *p*-value was lower than *p* = 0.0125 were the results considered statistically significant at α = 0.05. Tested groups were compared separately for PMMA and PET taking into account areas of remaining CS after 3,000 and 30,000 cycles. The level of significance was set to *p* = 0.05. Tested groups were compared separately for PMMA and PET taking into account areas of remaining CS after 3000 and 30,000 cycles.

## 3. Results and Discussion

### 3.1. Abrasion Resistance of Chitosan (CS) on Polyethylene Terephthalate (PET) Surfaces

The percentages of remaining CS on the PET surfaces after 3000 and 30,000 cycles of abrasion are shown in Table 3. In the test series II to VII, the medians and means of percentage of remaining CS coating were above 90% after 3000 cycles as well as after 30,000 cycles. In test series I (no Rocatec Plus sandblasting), the median dropped to 68.2% after 30,000 cycles with a minimum of remaining CS coating of 23.8%. The minima in all other test series were above 84%. Significant differences were found after 30,000 cycles of abrasion only (Table 4). The percentage of remaining CS in test series I differed significantly from series II. Further significant differences were found between test series IV and V, though their medians were above 90%.

### 3.2. Abrasion Resistance of CS on Polymethyl methacrylate (PMMA) Surfaces

The percentages of remaining CS on the PMMA surfaces after 3000 and 30,000 cycles of abrasion are shown in Table 5. Test series I (no Rocatec Plus sandblasting) showed medians of remaining CS coating of 85.5% after 3000 cycles and 55.2% after 30,000 cycles of abrasion. The minima were 69.9% (3000 cycles) and 0% (30,000 cycles). Test series II revealed median percentages of remaining CS of 88.2% after 3000 cycles and 73.1% after 30,000 cycles, whereas in test series IV the medians were 82.5% (3000 cycles) and 33.6% (30,000 cycles). Test series VII showed a median of remaining CS coating of 81.6% after 30,000 cycles of abrasion. The test series III, V and VI revealed medians above 90%. Significant differences were found after 3000 as well as after 30,000 cycles (Table 6).

### 3.3. Discussion

With exception of test series I after 3000 as well as after 30,000 cycles of abrasion (Table 3) a high resistance against abrasion was found on all PET surfaces. Within the PET test series II to VII, modifications in the workflow hardly influenced the abrasion resistance of CS after 3000 cycles (Table 4). Significant differences were found between test series IV *versus* V after 30,000 cycles of abrasion. However, the medians of test series II to VII were above 95%. Thus, within the limitations of this study, PET seems to be well suited to be coated with CS using the method described. For PET specimens, null hypothesis 1 (using/omitting Rocatec Plus) had to be rejected.

Also on PMMA surfaces (Table 5), abrasion-resistant bonds to CS could be achieved. However, the resistance against abrasion in test series II to VII seemed to be more heterogeneous compared to PET especially when 30,000 cycles of abrasion were conducted. The modifications in the workflow did partially impact the abrasion resistance in a significant manner (Table 6). It might therefore be concluded that the procedure to coat PMMA with CS is more technique sensitive as compared to PET. Hence, null hypothesis 1, as well as null hypothesis 5 (equal suitability for PMMA and PET for CS coating), had to be rejected.

On PMMA surfaces, the abrasion resistance after 30,000 cycles dropped significantly when the CS concentration was increased from 2% to 4% (test series II *versus* IV). This effect might be explained by an insufficient wetting of the sandblasted PMMA surface due to the high viscosity of the 4% acetic CS solution. However, the loss in abrasion resistance was compensated when the Rocatec Plus blasting pressure was increased from 2.8 to 4.0 bar (test series IV *versus* V). According to the manufacturer’s instructions, the Rocatec blast pressure should be at least 2.8 bar, but may be elevated to increase the particle impact energy. In our experiments, elevating the blast pressure above 4.0 bar led to intermittent disruptions of the continuous particle flow which might be due to turbulences at the blast nozzle. Thus, the authors recommend 4.0 bar Rocatec Plus sandblasting when a 4% acetic CS solution is applied. Based on these results, null hypothesis 2 (independence of abrasion resistance from CS concentration) and null hypothesis 3 (independence of abrasion resistance from blast pressure) had to be rejected.

**Figure 7:**
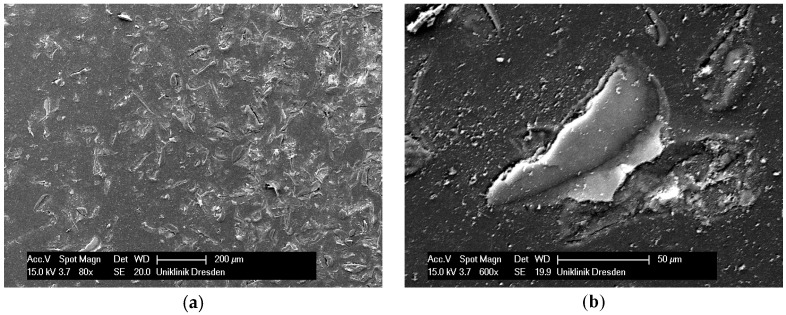
SEM picture of PET surface sandblasted at 2.8 bar Rocatec Pre and Rocatec Plus ((**a**) magnification 80×; (**b**): single impacted particle, magnification 600×).

**Figure 8:**
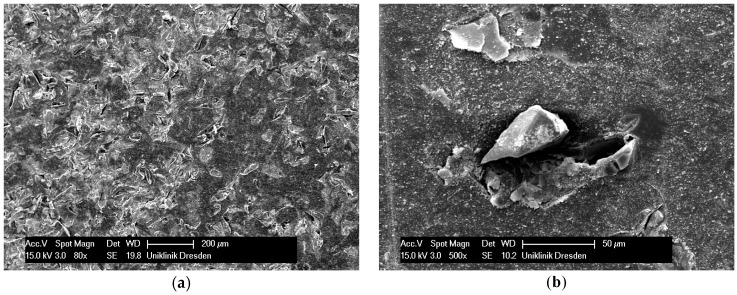
SEM picture of PMMA surface sandblasted at 2.8 bar Rocatec Pre and Rocatec Plus ((**a**) magnification 80×; (**b**) single impacted particle, magnification 500×).

The step of ultrasonic cleaning was conducted with the intention to remove loose particles from the resin surfaces after sandblasting with Rocatec Plus. With 4% CS acetate solution and 4.0 bar blast pressure, the medians of the remaining CS were 92.9% with ultrasonic cleaning and 81.6% and without ultrasonic cleaning (test series V *versus* VII). However, the differences were not significant. When sandblasting was done at 2.8 bar, the abrasion resistance dropped significantly when ultrasonic cleaning was carried out (test series IV *versus* VI). A reason might be a tighter bond of the impacted Rocatec Plus particles or their fragments to the resin material (Figure 7a,b and Figure 8a,b) when sandblasting was conducted at 4.0 bar, whereas at 2.8 bar, the looser impacted particles detach unintentionally during ultrasonic cleaning. Thus, with regard of a simple and cost-effective workflow, it seems to be justifiable to omit the step of ultrasonic cleaning. Null hypothesis 4 (independence of abrasion resistance from ultrasonic cleaning) also had to be rejected.

The 2% CS acetate solution exhibits a syrupy consistency that can be handled well during solution preparation as well as during the process of coating, whereas the high viscosity of the 4% CS acetate solution took the torque of the magnetic stirrer to its limit. However, for a clinical application, a 4% CS solution might be more useful because a higher amount of CS can be applied to the surface to be coated and the high viscosity may allow a better handling on curved surfaces of real-life denture bases or surgical splints. Within the method described in this study an abrasion-resistant coating could be achieved with 2% CS acetate solution as well as with 4% CS acetate solution.

Both the PET the PMMA test series I (Rocatec Pre only) performed worse compared to their corresponding test series II (Rocatec Pre and Rocatec Plus) although the grain sizes of Rocatec Pre and Plus are both 110 μm and preliminary experiments in this study revealed similar surface roughness measurements *R*_a_ after sandblasting with Rocatec Pre and Plus. Mean values from 10 measurements each revealed roughness profiles *R*_a_ of 3.04/2.98 μm (Rocatec Pre/Plus) on PMMA and 1.56/1.87 μm (Rocatec Pre/Plus) on PET surfaces (Hommel-Etamic W20, JENOPTIK Industrial Metrology, Villingen-Schwenningen Germany, probe Head TKU). However, without Rocatec Plus, sandblasting the bond of CS was substantially reduced on both PET and PMMA surfaces. It was concluded that micromechanical retention only cannot be a dominating factor of the bonding mechanism of CS to resin.

**Figure 9:**
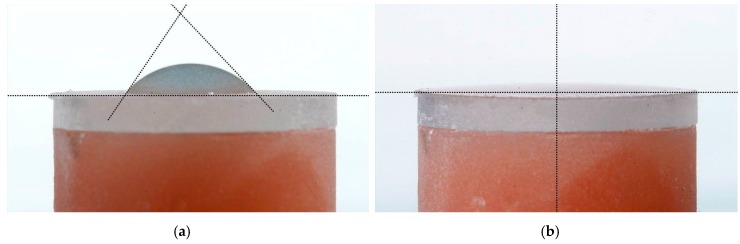
Sessile drop of water (20 μL) on PET surface sandblasted at 2.8 bar (**a**) Rocatec Pre only, contact angle 50°–60°; (**b**) Rocatec Pre and Rocatec Plus, contact angle 0°).

**Figure 10:**
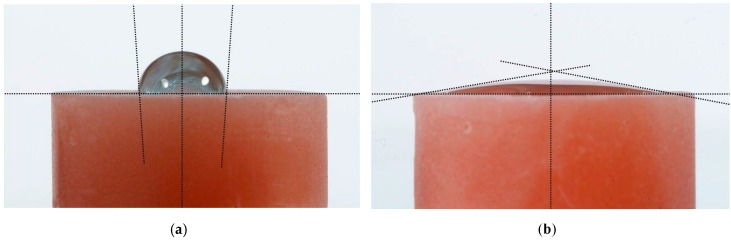
Sessile drop of water (20 μL) on PMMA surface sandblasted at 2.8 (**a**) Rocatec Pre only, contact angle 90°–100°; (**b**) Rocatec Pre and Rocatec Plus, contact angle 5° to 10°.

**Figure 11:**
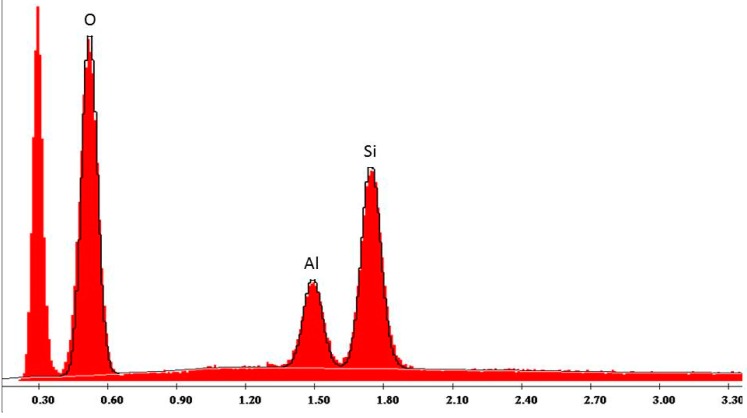
EDAX Analysis of PMMA surface sandblasted with Rocatec Pre and Rocatec Plus (110 μm silicate-modified aluminum oxide, 2.8 bar).

**Figure 12:**
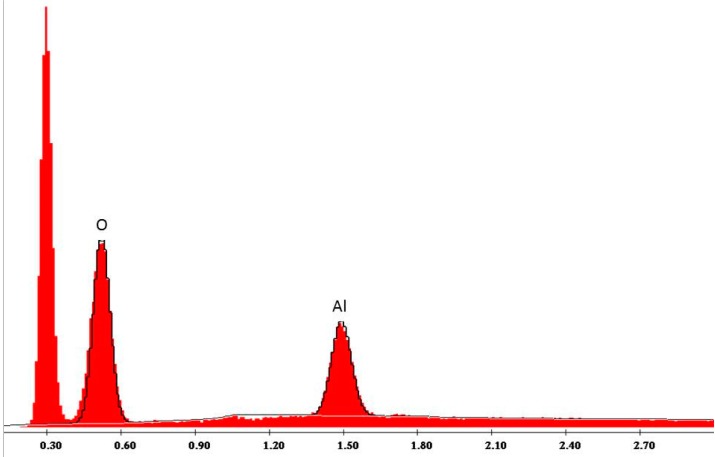
EDAX Analysis of PMMA surface sandblasted with Rocatec Pre only (110 μm aluminum oxide, 2.8 bar).

The main application of the Rocatec system in dental technology is the tribo-chemical silicate coating of dental ceramics, resins or metals to achieve a chemical bond to adhesive resins in dental prostheses through an organic silane coupling agent [10,11]. Sandblasting of PMMA or PET with Rocatec Plus generates hydrophilic surfaces. For illustration, cylindrical PET and PMMA specimens (13 mm diameter, 6 mm height) were sandblasted at 2.8 bar and a distance of 3 cm either with Rocatec Pre only or with Rocatec Pre and Rocatec Plus. Distilled water was stained with methylene blue and one drop carefully applied to the sandblasted surface using a measuring pipette (Biomaster 20 μL, Eppendorf AG, Hamburg, Germany). On the Rocatec Pre-sandblasted surfaces the higher contact angles indicated a poor wettability (Figure 9a and Figure 10a) while the drops immediately spread across the surface after Rocatec Plus sandblasting (Figure 9b and Figure 10b). Furthermore, EDAX analysis revealed the presence of silicon after sandblasting with Rocatec Plus (Figure 11), while no silicon was detected on resin surfaces when sandblasted with Rocatec Pre only (Figure 12). Obviously, wettability of resin surfaces is a key factor to bond CS and can be achieved with different technologies [12].

Reviewing the literature, the adhesion mechanism of PMMA or PET to CS remains inconclusive. Preliminary experiments in this study showed that neutralization of the acetic CS solution with NaOH and thus an alkaline environment prior to the drying process inhibited a bond between CS and the resin surface whereas an abrasion-resistant bond was given when neutralization with NaOH was accomplished after the step of drying. In an acetic solution the amino groups of the chitosan molecules are positively charged (NH_3_^+^) and may be attracted by the electronegativity of the hydroxyl groups at the silicate surfaces. The dehydration process detracts the water from the acetic CS solution, leaving a solid CS layer on the resin surfaces. There are several mechanisms proposed for a potential coupling between chitosan and silicate [13]. Both primary amino and hydroxyl groups in the CS molecules are potential coupling points to covalently bond to silicate during a dehydration process [14,15]. One option might be an ionic bond of positively charged amino groups and negatively charged hydroxyl ions [16,17]. Another hypothesis is a process of condensation of hydrolyzed silicate groups with hydroxyl groups from CS resulting in a covalently bonded chitosan–silicate hybrid layer [18].

## 4. Conclusions

This study introduced a simple procedure to coat PMMA and PET with CS comprising five steps:

1. Sandblasting of the surfaces to be CS coated with Rocatec Pre and Rocatec Plus (2.8 to 4.0 bar blast pressure)
2. Coating with acetic CS solution (2% to 4% CS)
3. Drying of the CS solution for 120 min at 45 °C.
4. Neutralization of the precipitated CS in mol NaOH solution for 10 min
5. Washing in distilled water.

The procedure allows a very abrasion-resistant bond of CS to PET. The change of parameters as described in this study (blast pressure 2.8 bar/4.0 bar, CS concentration 2%/4%) has no impact on abrasion resistance. Bonding CS to PMMA is more technique sensitive. When 4% acetic CS solution is applied, blast pressure should be 4 bar; when 4% acetic CS solution is applied, the blast pressure should be 2.8 bar. The procedure requires equipment normally found in standard dental laboratories. The abrasion-resistant bond to PMMA and PET does not need any organic coupling agents, primers or bonders that might induce allergic responses or other adverse effects to the human body. Rocatec Plus sand contains aluminum oxide and silicate and has long been used in dental technology. The hemostatic and antimicrobial properties of the CS coatings in this study will be subject to clinical research. However, additional preclinical data, such as on standardized coating thickness and the potential effect of dental disinfectants are needed before approval can be obtained from an ethics committee to conduct a clinical study. Furthermore the technology described may useful also outside dentistry.

## Figures and Tables

**Table 1:** Variations of steps 1 to 3 of the basic procedure to coat PMMA (*n* = 10 each) and PET (*n* = 10 each) with chitosan. Steps 4 to 6 of the basic procedure were not changed.

Test series	Steps of basic procedure
I	2.8 bar sandblasting Rocatec Pre/no Rocatec Plus ultrasonic cleaning 3.2% chitosan acetate
II	2.8 bar sandblasting Rocatec Pre/2.8 bar Rocatec Plus ultrasonic cleaning 2% chitosan acetate
III	2.8 bar sandblasting Rocatec Pre/4.0 bar Rocatec Plus ultrasonic cleaning 3.2% chitosan acetate
IV	2.8 bar sandblasting Rocatec Pre/2.8 bar Rocatec Plus ultrasonic cleaning 4% chitosan acetate
V	2.8 bar sandblasting Rocatec Pre/4.0 bar Rocatec Plus ultrasonic cleaning 4% chitosan acetate
VI	2.8 bar sandblasting Rocatec Pre/2.8 bar Rocatec Plus ultrasonic cleaning omitted 4% chitosan acetate
VII	2.8 bar sandblasting Rocatec Pre/4.0 bar Rocatec Plus ultrasonic cleaning omitted 4% chitosan acetate

**Table 2:** Composition of artificial saliva.

1.	82.93% Aqua dest
2.	12.50% Hydroxyethyl cellulose (4%)
3.	4.28% Sorbitol solution (70%)
4.	0.12% Potassium chloride
5.	0.08% Sodium chloride
6.	0.06% Sodium monohydrogenphosphate 12 H_2_O
7.	0.02% Calcium chloride 2 H_2_O
8.	0.01% Magnesium chloride 6 H_2_O
9.	Preservative: Propyl 4-Hydroxybenzoate

**Table 3:** Percentage of remaining chitosan on PET specimens after abrasion tests (*n* = 10).

**Percentage of remaining chitosan after 3000 cycles of abrasion**						
**Test Series**	**Min**	**1. Quartile**	**Median**	**Mean**	**3. Quartile**	**Max**
I	73.9	98.0	99.8	95.1	100.0	100.0
II	99.1	99.9	100.0	99.9	100.0	100.0
III	99.0	99.9	100.0	99.9	100.0	100.0
IV	98.4	99.5	99.6	99.8	100.0	100.0
V	93.5	96.3	96.9	98.6	99.5	100.0
VI	99.4	100.0	100.0	100.0	100.0	100.0
VII	95.9	98.5	99.2	99.5	99.6	100.0
**Percentage of remaining chitosan after 30,000 cycles of abrasion**						
**Test Series**	**Min**	**1. Quartile**	**Median**	**Mean**	**3. Quartile**	**Max**
I	23.8	45.3	68.2	61.8	79.4	93.3
II	88.8	98.2	99.2	97.4	100.0	100.0
III	97.7	99.6	100.0	99.6	100.0	100.0
IV	91.6	98.3	98.8	98.3	100.0	100.0
V	84.2	92.6	95.9	94.5	97.7	99.2
VI	91.8	99.6	100.0	98.6	100.0	100.0
VII	93.2	97.1	99.0	98.1	99.7	100.0

**Table 4:** Statistical analysis (*U*-Test, *p* = 0.05 with Bonferroni correction, *p* = 0.0125) of test series with respect to remaining chitosan coating on PET.

**3,000 cycles of abrasion**			
**Test series**	***U***	***p***	**Bonferroni-corrected significance**
I *versus* II	23.0	0.04516	–
II *versus* III	49.5	1.00000	–
II *versus* IV	36.0	0.30749	–
IV *versus* V	37.0	0.34471	–
IV *versus* VI	39.0	0.42736	–
IV *versus* VII	36.0	0.30749	–
V *versus* VII	43.0	0.62318	–
VI *versus* VII	28.0	0.10411	–
VI *versus* III	45.0	0.73373	–
**30,000 cycles of abrasion**			
**Test series**	***U***	***p***	**Bonferroni-corrected significance**
I *versus* II	2.0	0.00033	*
II *versus* III	36.0	0.30749	–
II *versus* IV	47.0	0.85011	–
IV *versus* V	14.0	0.00729	*
IV *versus* VI	36.5	0.32575	–
IV *versus* VII	45.0	0.73373	–
V *versus* VII	19.5	0.02334	–
VI *versus* VII	32.0	0.18588	–
VI *versus* III	46.5	0.82060	–

* statistically significant with Bonferroni correction.

**Table 5:** Percentage of remaining chitosan on PMMA specimens after abrasion tests (*n* = 10).

**Percentage of remaining chitosan after 3,000 cycles of abrasion**						
**Test Series**	**Min**	**1. Quartile**	**Median**	**Mean**	**3. Quartile**	**Max**
**I**	69.0	79.7	85.5	85.3	93.2	97.1
**II**	47.1	80.8	88.2	85.2	95.7	100.0
**III**	96.9	99.6	99.9	99.5	100.0	100.0
**IV**	50.6	71.1	82.4	79.6	94.9	98.3
**V**	90.5	96.9	97.7	97.0	99.8	100.0
**VI**	92.7	99.6	99.8	98.7	99.9	100.0
**VII**	90.0	96.4	98.4	97.4	99.7	100.0
**Percentage of remaining chitosan after 30,000 cycles of abrasion**						
**Test Series**	**Min**	**1. Quartile**	**Median**	**Mean**	**3. Quartile**	**Max**
**I**	0.0	48.2	55.2	51.7	64.5	68.6
**II**	3.6	56.9	73.1	66.6	81.4	95.7
**III**	89.1	96.6	99.0	97.4	99.1	99.8
**IV**	5.0	13.2	33.6	32.9	49.5	70.1
**V**	73.5	79.5	92.9	87.6	94.6	95.8
**VI**	71.4	91.9	98.0	94.1	99.3	100.0
**VII**	64.9	71.9	81.6	80.3	89.9	91.8

**Table 6:** Statistical analysis (*U*-test, *p* = 0.05 with Bonferroni correction, *p* = 0.0125) of test series with respect to remaining chitosan coating on PMMA.

**3,000 cycles of abrasion**			
**Test series**	***U***	***p***	**Bonferroni-corrected significance**
I *versus* II	41.0	0.52052	–
II *versus* III	16.0	0.01133	*
II *versus* IV	39.0	0.42736	–
IV *versus* V	19.0	0.02114	–
IV *versus* VI	9.0	0.00220	*
IV *versus* VII	10.0	0.00283	*
V *versus* VII	37.0	0.34471	–
VI *versus* VII	48.5	0.93974	–
VI *versus* III	29.0	0.12123	–
**30,000 cycles of abrasion**			
**Test series**	***U***	***p***	**Bonferroni-corrected significance**
I *versus* II	22.0	0.03764	–
II *versus* III	4.0	0.00058	*
II *versus* IV	13.0	0.00580	*
IV *versus* V	0.0	0.00018	*
IV *versus* VI	0.0	0.00018	*
IV *versus* VII	2.0	0.00033	*
V *versus* VII	25.0	0.06402	–
VI *versus* VII	14.0	0.00729	*
VI *versus* III	36.0	0.30749	–

*statistically significant with Bonferroni correction

## Abbreviations

The following abbreviations are used in this manuscript:

CS: chitosan
PMMA: polymethyl methacrylate
PET: polyethylene terephthalate

## References

[1] Rinaudo M. (2006). Chitin and chitosan: Properties and applications. Prog. Polym. Sci..

[2] Kumar M.N.V.R. (2000). A review of chitin and chitosan applications. React. Func. Polym..

[3] Zhao Y., Park R.D., Muzzarelli R.A.A. (2010). Chitin deacetylases: Properties and applications. Mar. Drugs.

[4] Kumar M.N., Muzzarelli R.A.A., Muzzarelli C., Sashiwa H., Domb A.J. (2004). Chitosan chemistry and pharmaceutical perspectives. Chem. Rev..

[5] Muzzarelli R.A.A., Muzzarelli C. (2005). Chitosan chemistry: Relevance to the biomedical sciences. Adv. Polym. Sci..

[6] Muzzarelli R.A.A. (2009). Chitins and chitosans for the repair of wounded skin, nerve, cartilage and bone. Carbohydr. Polym..

[7] Patel M.P., Patel R.R., Patel J.K. (2010). Chitosan mediated targeted drug delivery system: A review. J. Pharm. Pharmaceut. Sci..

[8] Salerno C., Pascale M., Contaldo M., Esposito V., Busciolano M., Milillo L., Guida A., Petruzzi M., Serpico R. (2011). Candida-associated denture stomatitis. Med. Oral. Patol. Oral. Cir. Bucal.

[9] Gendreau L., Loewy Z.G. (2011). Epidemiology and etiology of denture stomatitis. J. Prosthodontics.

[10] Kern M., van Thompson V.P. (1994). Sandblasting and silica coating of a glass-infiltrated alumina ceramic: Volume loss, morphology, and changes in the surface composition. J. Prosthet. Dent..

[11] Robina C., Scherrera S.S., Wiskotta H.W.A., de Rijkb W.G., Belsera U.C. (2002). Weibull parameters of composite resin bond strengths to porcelain and noble alloy using the Rocatec system. Dent. Mater..

[12] Massouda D., Cotts P. (2006). Attachment of Chitosan to Surfaces Using Rehydration Process. US Patent.

[13] Connell L.S., Romer F., Suarez M., Valliant E.M., Ziyu Zhang Z., Lee P.D., Smith M.E., Hannab J.V., Jones J.R. (2014). Chemical characterisation and fabrication of chitosan–silica hybrid scaffolds with 3-glycidoxypropyltrimethoxysilane. J. Mater. Chem. B.

[14] Liua Y.L., Sua Y.H., Leeb K.R., Laia J.Y. (2005). Crosslinked organic–inorganic hybrid chitosan membranes for pervaporation dehydration of isopropanol–water mixtures with a long-term stability. J. Memb. Sci..

[15] Liu Y.L., Su Y.H., Lai J.Y. (2004). *In situ* crosslinking of chitosan and formation of chitosan–silica hybrid membranes with using γ-glycidoxypropyltrimethoxysilane as a crosslinking agent. Polymer.

[16] Shirosaki Y., Tsuru K., Hayakawa S., Osaka A., Lopes M.A., Santos J., Costa M., Fernandes M. (2009). Physical, chemical and *in vitro* biological profile of chitosan hybrid membrane as a function of organosiloxane concentration. Acta Biomater..

[17] Rashidovaa S.S., Shakarovaa D.S., Ruzimuradova O.N., Satubaldievaa D.T., Zalyalievaa S.V., Shpigunb O.A., Varlamovc V.P., Kabulova B.D. (2004). Bionanocompositional chitosan–silica sorbent for liquid chromatography. J. Chromatogr. B.

[18] Al-Sagheer F., Muslim J. (2010). Thermal and mechanical properties of chitosan/SiO_2_ hybrid composites. J. Nanomater..

